# Trumps Wirtschaftspolitik und der Corona-Schock - Perspektiven für die USA

**DOI:** 10.1007/s10273-020-2786-0

**Published:** 2020-11-20

**Authors:** Paul J. J. Welfens

**Affiliations:** European Institute for International Economic Relations, University of Wuppertal, Rainer-Gruenter-Str. 21, 42119 Wuppertal, Deutschland

**Keywords:** E61, F00, I18

## Abstract

US-Präsident Donald Trump hat am 3. November 2020 zwar eine Niederlage erfahren, dennoch könnte der seit 2016 politisch in den USA herrschende Populismus als Mischung aus neuem Nationalismus und Protektionismus für einige Jahre andauern. Die US-Wahlen fanden unter besonderen Bedingungen statt: so löste der Corona-Schock einen weltweiten Wirtschaftsschock aus.

## Abstract (en)

The United States is characterised by a structural populism problem that persists even without Trump as president. Its economic policy is fundamentally contradictory. The US fiscal and deficit policy under Trump was considered risky even before the corona shock, while the aggressive US trade policy is a self-inflicted wound. If we look at effective lifetime income, Germany, France and the United States are on an equal footing. This is due in part to the lag in life expectancy in the US and the much higher health care costs - relative to GDP - in the American system. A transatlantic dialogue on reform would be worthwhile after the corona shock of 2020.

In Bezug auf die Wahl in den USA stellen sich verschiedene Fragen. Was hat die Trump-Administration von 2016 bis 2020 ökonomisch erreicht? Wie fällt die Bilanz der US-Epidemiepolitik aus? Und welche Probleme haben sich für den Westen insgesamt seit 2016 mit den beiden „Politikschocks" Brexit-Mehrheit bei der britischen EU-Volksabstimmung und Trump als Präsident in den USA ergeben? Schließlich auch, was waren die Gründe für die Expansion des politischen Populismus? Und dies jenseits einer verbreiteten Enttäuschung über die politische Elite im Umgang mit der US-Bankenkrise 2008/2009, die auch eine transatlantische Wirtschaftskrise war. Damals entstand vielfach der Eindruck, dass für die Rettung von Großbanken gewaltige Summen aus der Staatskasse verfügbar gemacht wurden, während von der Banken- und Wirtschaftskrise betroffenen Arbeitnehmern nur relativ wenig Hilfspakete angeboten wurden.

**Prof. Dr. Paul J. J. Welfens** ist Präsident des Europäischen Instituts für Internationale Wirtschaftsbeziehungen (EIIW) an der Bergischen Universität Wuppertal und Jean Monnet Professor für European Economic Integration, Chair for Macroeconomics. Er war Alfred Grosser Professor am Sciences Po, Paris und ist Non-resident Senior Research Fellow am AICGS/ Johns Hopkins University, Washington DC und zudem Research Fellow am IZA, Bonn.

Als Trump im November 2016 – aus Sicht der meisten US-Medien – überraschend zum 45. Präsidenten der USA gewählt wurde und dann im Januar 2017 das Amt von seinem Vorgänger Barack Obama übernahm, setzte er wirtschaftspolitisch auf fünf Richtungsentscheidungen:

- Eine Steuerreform, die insbesondere eine Senkung der Körperschaftsteuersätze von 35 % auf 21 % brachte, aber auch Anreize dafür, im Ausland geparkte Gewinne von US-Multis zu günstigen Steuerbedingungen in die USA zurückzuführen;
- eine verstärkte Orientierung der internationalen US-Handelspolitik hin auf nationale Ziele; Trump versprach schon im Wahlkampf 2016, dass er neue Deals mit vorteilhafteren Bedingungen in Handelsverträgen abschließen wolle – und damit sollte dann auch die Defizitquote der US-Handelsbilanz schrumpfen. Der Vertrag über die Freihandelszone USA-Kanada-Mexiko wurde neu verhandelt und läuft mit leicht modifizierten Bedingungen, wobei unter anderem eine höhere Mindestwertschöpfungsquote in Mexiko und Kanada als bisher für einen freien US-Marktzutritt vorgesehen ist.
- Eine Deregulierung der Wirtschaft, insbesondere auch im Energie- und Umweltbereich sowie höhere Ausgaben für Infrastruktur;
- einen Ausstieg aus dem Pariser UN-Klimaabkommen, was die US-Wirtschaft vor ökologisch unnötigen Belastungen bewahren sollte;
- eine Schwächung internationaler Organisationen bzw. des Multilateralismus wie etwa in Bezug auf die Rolle der Welthandelsorganisation und der WHO (letztere mit Reformbedarf).

Während die Schwächung multilateraler Organisationen ideologisch eine durchaus alte Verankerung in Teilen der Republikanischen Partei hat und Steuersenkungen bei jedem republikanischen Präsidenten zum Anfang der ersten Amtszeit üblicher Politikbestandteil sind, ist in der Handelspolitik die Tradition der Republikaner eher auf Freihandel gerichtet – mit Ausnahme der Amtszeit von Ronald Reagan, dem in den 1980er Jahren vor allem hohe bilaterale Leistungsbilanzüberschüsse Japans Probleme mit Blick auf die US-Leistungsbilanzposition beschert hatten. In der Klimaschutzpolitik geht es um ein relativ neues Thema, bei dem jedoch führende US-Wissenschaftler tatsächlich ernsten Handlungsbedarf auf mittlere und lange Frist sehen. Die 2017 durchgeführte Steuerreform brachte nicht nur eine Senkung der Körperschaftsteuersätze, sondern auch verstärkte Exportanreize. In besonderer Weise suchten die USA hierbei offenbar eine indirekte Exportförderung zu realisieren und damit der indirekten Exportförderung der EU-Länder und Chinas entgegenzuwirken, die anders als die USA eine Mehrwertsteuer auf Importe erheben.

### Fiskal- und Staatsschuldenpolitik der USA

Deregulierungsdruck und expansive Fiskalpolitik, kombiniert mit Steuersenkungen, gehören zum US-Wirtschaftsliberalismus und sind durchaus Teil der Politik verschiedener US-Präsidenten – insbesondere der Republikanischen Partei – gewesen. Die Analyse von Frankel (2006) weist auf die Lehren aus der US-Geschichte der Wirtschaftspolitik hin, wonach Republikanische Präsidenten stets mit Militärausgabenerhöhung und Steuerminderungspolitik kurz nach dem Amtsantritt beginnen und dann mit einer hohen Budget-Defizitquote für einige Zeit zurechtkommen müssen. Trump machte das jedoch stärker als frühere Administrationen und verzeichnete auch im Wirtschaftsaufschwung 2018/2019 hohe Defizitquoten, sodass für den Fall einer Rezession schon mit außergewöhnlich hohen Defizitquoten zu rechnen war – mit dem Corona-Schock-Jahr 2020 wurden dann alle Befürchtungen noch deutlich übertroffen: Die Defizitquote überstieg sogar die 11 % aus der Bankenkrise 2008/2009.

Dabei war, wenig erstaunlich, auch ein erheblicher Anstieg der Schuldenquote zu verzeichnen, die jedoch effektiv wegen des enormen Ankaufs von US-Staatsanleihen durch die US-Zentralbank in der Prognose des IWF (2020b) noch gedämpft ausfiel. Wenn die US-Zentralbank etwa Bundes-Anleihen in Höhe von 30 % des BIP hält, was ungewöhnlich hoch ist, so bedeutet das bei einem Zinssatz von 3 %, dass der US-Bundesstaat eine Zinsausgabenquote (Zinsausgaben relativ zum BIP) von 1 % erreicht, die er an sich selbst zahlt: zunächst an das Federal Reserve System, da die Fed US-Staatsanleihen massiv angekauft hat. Die staatlichen Zinszahlungen an die Fed gehen allerdings über erhöhte Notenbankgewinne, die an den US-Staat abzuführen sind, an den Staat zurück. Allerdings ist der Zinssatz auf US-Staatsanleihen langfristig nominal und real gesunken – unter der Trump-Administration aber real 2018 wieder leicht gestiegen. Das US Congressional Budget Office (CBO, 2020) hatte noch vor dem Corona-Schock errechnet, dass eine Fortsetzung der Budget-Politik Trumps bis 2050 die US-Staatsschuldenquote von knapp 100 % (2019) auf 180 % ansteigen lassen würde.

Das bei Standard & Poor's schon in der Banken- und Wirtschaftskrise 2008/2009 herabgesetzte Rating bei US-Staatsanleihen von AAA auf AA wäre bei einer solchen Entwicklung wohl schon Ende der dritten Dekade des 21. Jahrhunderts im B-Bereich, was für die US-Wirtschaft und den US-Dollar eine deutliche Schwächung brächte. Dass der US-Dollar gegenüber dem Euro im Sommer 2020 eine Abwertung erlebte, dürfte auf die Wahrnehmung internationaler Investoren zurückzuführen sein, dass die USA unter Trump – und erst recht im Corona-Schock-Jahr 2020 – kaum noch internationale Führungsqualität in der Wirtschaftspolitik zeigen, während zugleich die US-Staatsschuldenquote enorm ansteigt: stärker als in der Eurozone. Demnach ist beim gegenwärtigen US-Politikmix mittelfristig auch ein ausgeprägteres Quantitative Easing der Geldpolitik in den USA (Druck der Politik auf die Fed in diese Richtung) – im Vergleich zur Eurozone – zu erwarten, was den Abwertungsdruck auf den US-Dollar 2020 erklären kann.

Wenn man die IWF-Prognose von 2019 und 2020 für die USA vergleicht (vgl. Tabelle 1 bzw. Tabelle 2), so weist der IWF für 2024 in seiner Prognose von 2020 deutlich veränderte Zahlen gegenüber der Prognose von 2019 aus. Dabei hat sich für 2020 die Arbeitslosenquote in der Prognose von 2020 gegenüber der Erwartung von 2019 fast verdreifacht; der erwartete Schuldenbestand relativ zum BIP für 2024 hat sich um fast 25 Prozentpunkte erhöht – eine Erhöhung um gut ein Viertel gegenüber der Prognose von 2019. Die Outputlücke dreht in der aktuellen IWF-Prognose ab 2020 für mehrere Jahre ins Negative. Der Corona-Wirtschaftsschock ist also massiv, wie dies auch der IWF (2020c) im World Economic Outlook/Update im Juni 2020 für die Weltwirtschaft ausgewiesen hat: Es wird erwartet, dass die realen Einkommen weltweit 2020 um rund 4,5 % schrumpfen.

### Quantitative Easing und Staatsschuldenquote

Die USA haben 2009/2010 – und nachfolgend Großbritannien und die Eurozone – zur Überwindung der Transatlantischen Bankenkrise stark auf Quantitative Easing (QE) gesetzt; auf eine umfassende expansive Offenmarktpoli-

Tabelle 1

**Prognose ausgewählter Wirtschaftsindikatoren der USA, 2019**

| | | Prognose | | | | | |
|---|---|---|---|---|---|---|---|
| | 2018 | 2019 | 2020 | 2021 | 2022 | 2023 | 2024 |
| Reales BIP, % zum Vorjahr | 2,9 | 2,6 | 1,9 | 1,8 | 1,7 | 1,6 | 1,6 |
| Reales BIP, Q4/Q4, % | 3,0 | 2,3 | 1,9 | 1,7 | 1,7 | 1,6 | 1,6 |
| Outputlücke, % des potenziellen BIP | 1,1 | 1,7 | 1,7 | 1,6 | 1,6 | 1,5 | 1,4 |
| Arbeitslosenquote, Q4, % | 3,9 | 3,6 | 3,5 | 3,6 | 3,7 | 3,8 | 3,8 |
| Leistungsbilanzsaldo, % des BIP | -2,3 | -2,1 | -2,5 | -2,7 | -2,6 | -2,5 | -2,4 |
| Leitzins, Ende der Periode, % | 1,8 | 2,4 | 2,6 | 2,9 | 2,9 | 2,9 | 2,8 |
| Staatsanleihen mit zehnjähriger Laufzeit, Q4, % | 2,9 | 2,7 | 2,9 | 3,1 | 3,2 | 3,2 | 3,2 |
| Inflation, PCE[1], Q4/Q4, % | 1,9 | 2,1 | 2,2 | 2,0 | 2,0 | 2,0 | 2,0 |
| Kerninflation (PCE, Q4/Q4), % | 1,9 | 1,8 | 2,1 | 2,1 | 2,0 | 2,0 | 2,0 |
| Budget-Defizitquote, % des BIP | -3,9 | -4,2 | -4,0 | -4,0 | -4,3 | -4,1 | -3,8 |
| Staatsschuldenbestand von Anlegern gehalten, % des BIP | 77,8 | 78,7 | 79,6 | 80,6 | 82,0 | 83,3 | 84,2 |

[1] Personal Consumption Expenditures.

Tabelle 2

**Prognose ausgewählter Wirtschaftsindikatoren der USA, 2020**

| | | Prognose | | | | | | |
|---|---|---|---|---|---|---|---|---|
| | 2018 | 2019 | 2020 | 2021 | 2022 | 2023 | 2024 | 2025 |
| Reales BIP, % zum Vorjahr | 2,9 | 2,3 | -6,6 | 3,9 | 3,3 | 2,3 | 1,9 | 1,8 |
| Reales BIP, Q4/Q4, % | 2,5 | 2,3 | -6,9 | 5,1 | 2,8 | 2,0 | 1,9 | 1,8 |
| Outputlücke, % des potenziellen BIP | 0,2 | 0,9 | -4,9 | -1,8 | -0,6 | -0,4 | -0,4 | -0,5 |
| Arbeitslosenquote, Q4, % | 3,8 | 3,5 | 9,7 | 7,4 | 5,7 | 4,6 | 4,3 | 4,2 |
| Leistungsbilanzsaldo, % des BIP | -2,2 | -2,2 | -2,2 | -2,1 | -2,1 | -2,1 | -2,0 | -2,0 |
| Leitzins, Ende der Periode, % | 2,2 | 1,7 | 0,1 | 0,1 | 0,1 | 0,1 | 0,1 | 0,1 |
| Staatsanleihen mit zehnjähriger Laufzeit, Q4, % | 3,0 | 1,8 | 0,8 | 1,0 | 1,5 | 1,7 | 1,8 | 1,8 |
| Inflation, PCE[1], Q4/Q4, % | 1,9 | 1,4 | 0,7 | 2,1 | 2,0 | 2,0 | 2,0 | 2,0 |
| Kerninflation (PCE, Q4/Q4), % | 1,9 | 1,6 | 0,8 | 1,8 | 1,9 | 1,9 | 1,9 | 1,9 |
| Budget-Defizitquote, % des BIP | -3,8 | -4,6 | -18,0 | -10,4 | -5,4 | -4,5 | -4,7 | -4,9 |
| Staatsschuldenbestand von Anlegern gehalten, % des BIP | 77,4 | 79,2 | 99,6 | 107,4 | 106,8 | 106,8 | 107,4 | 108,3 |

[1] Personal Consumption Expenditures.

tik (mit Erhöhung der Geldmenge via Ankauf von Staatsschuldtiteln) in einem Umfeld mit einem Notenbankzinssatz nahe null. Ähnliches geschieht nun auch, wobei der Umfang staatlicher Anleihen im Bestand der Zentralbank rund 30 % beträgt, wie man aus dem Vergleich von Brutto-Staatsschuldenquote und den Staatsschulden privater Anleger relativ zum BIP erkennen kann. Staatsschulden in der Hand privater Anleger – relativ zum BIP – ergeben sich aus der Brutto-Staatsschuldenquote, nachdem man die Staatsanleihen in der Bilanz der Zentralbank abgezogen hat; für 2019 weist Tabelle 3 eine staatliche Schuldenquote von 109 % (2019) in den USA aus, während als Staatsschuldenbestand – relativ zum BIP – bei privaten Anlegern in Tabelle 2 nur 99,6 % angegeben werden: 2019 waren also 9,4 % Staatsschuldenquote bei der Zentralbank gehalten, wobei die Prognosewerte für 2021 einen Anstieg der „Notenbank-Staatsschuldenquote“ auf 24,5 % anzeigen. Das ist ein relativ hoher Anteil der Staatsschuld, der dem US-Bundesstaat auch erhebliche Zinsersparnisse einbringt. Wenn der Staat 1 % faktisch an Zinsausgabenquote einspart, so kann dadurch die durchschnittliche Steuerquote um einen Prozentpunkt geringer ausfallen als sonst. Das erhöht das Niveau des Wachstumspfades in einem Solow-Wachstumsmodell.

Man kann im Kontext des Branson-Modells zeigen, dass Quantitative Easing zu einer nominalen – und vorübergehend auch realen Währungsabwertung – sowie zu einer nominalen und zeitweise auch realen Zinssenkung führt, sodass diese Geldpolitik den Außenbeitrag und die Investitionen stimuliert. Von einer US-Dollar-Abwertung gegenüber dem Britischen Pfund und Euro sollte allerdings 2020 im Kontext von Quantitative Easing wenig zu sehen sein, da auch die Bank of England und die EZB eine QE-Geldpolitik eingeschlagen haben, also die „europäische Verzögerung“ bei QE nicht der Transatlantischen Bankenkrise entspricht.

Die vom IWF für 2020 laut Fiscal Monitor erwartete Budget-Defizitquote der USA von 15,5 % ist noch deutlich höher als die in der Eurozone, wobei die Defizitquote 2021 mit fast 9 % in den USA relativ hoch ausfällt, sodass die US-Schuldenquote auch mittelfristig weiter steigen wird (vgl. Tabelle 3 und Tabelle 4). Um mehr als 3 Prozentpunkte kann man erfahrungsgemäß in einem Jahr die Defizitquote nicht senken, sodass die US-Schuldenquote 2023 etwa 34 Prozentpunkte höher stehen dürfte als 2019, was den US-Dollar tendenziell schwächt. In der Eurozone ist Italien bei der Schuldenquote vor Probleme gestellt, da diese im Verlauf von 2020 laut Fiscal Monitor-Prognose des IWF auf 156 % – plus 21 Prozentpunkte gegenüber 2019 – ansteigen wird. Erfahrungsgemäß kann der Staat die Defizitquote pro Jahr um nicht mehr als 3 Prozentpunkte vermindern, sodass Italiens Defizitquote von fast

Tabelle 3
**Staatliche Defizitquoten, ausgewählte Länder**
in % des BIP

| Finanzierungs-saldoposition | 1990 | 1995 | 2000 | 2005 | 2010 | 2015 | 2019 | 2020[a] | 2021[a] |
|---|---|---|---|---|---|---|---|---|---|
| Deutschland | | -9,43 | -1,58 | -3,32 | -4,38 | 0,94 | 1,45 | -5,52 | -1,19 |
| Schweiz | 0,00 | -1,87 | 0,42 | -0,67 | 0,36 | 0,64 | 0,89 | -5,06 | -1,90 |
| Südkorea | | 2,24 | 4,05 | 0,82 | 1,47 | 0,52 | 0,86 | -1,84 | -1,57 |
| Italien | -11,10 | -7,21 | -2,42 | -4,08 | -4,24 | -2,55 | -1,64 | -8,34 | -3,47 |
| Großbritannien | -1,39 | -4,97 | 1,36 | -3,15 | -9,28 | -4,59 | -2,08 | -8,31 | -5,47 |
| Spanien | -3,91 | -6,80 | -1,16 | 1,23 | -9,53 | -5,18 | -2,64 | -9,51 | -6,66 |
| Japan | 1,99 | -4,39 | -8,25 | -4,96 | -9,53 | -3,81 | -2,82 | -7,09 | -2,06 |
| Frankreich | -2,43 | -5,11 | -1,32 | -3,36 | -6,89 | -3,63 | -3,02 | -9,17 | -6,24 |
| Australien | -0,23 | -2,61 | 1,25 | 1,71 | -5,13 | -2,80 | -3,73 | -9,73 | -7,34 |
| USA | | | | -3,07 | -11,02 | -3,57 | -5,78 | -15,45 | -8,64 |

[a] Prognose.

Quelle: IWF (2020a), Fiscal Monitor April 2020, Datenbank.

21 % (2020) für die sechs Folgejahre nochmals einen Anstieg der Schuldenquote um etwa 60 % erwarten lässt. Wegen der EU-Transfers aus dem 390 Mrd. Euro-Gesamt-Transfer-Paket der EU-Kommission vom Sommer 2020 und einem 2021 denkbar starken Aufschwung sowie EZB-Ankäufen von Staatsanleihen mag Italiens Schuldenquote bis 2026 beim Publikum nur um weitere 40 % ansteigen, aber mit fast 200 % Staatsschuldenquote in den Händen privater Anleger liefe Italien auf mittlere Sicht einem Misstrauensvotum durch die Finanzmärkte entgegen. Das Problem wird dabei nur wenig dadurch gemildert, dass Italiens Leistungsbilanzüberschüsse es mit sich bringen, dass überwiegend Anleger aus Italien – und einigen EU-Ländern – italienische Staatsanleihen haben (eine denkbare Vermögensabgabe wäre sicher politisch unpopulär). Italiens Schuldenquote wäre 2023 wohl ähnlich schlecht wie bei Griechenland 2012; und ein mittelfristiges Sinken des Risikoappetits von Investoren könnte dann zu massiv steigenden Risikoprämien bei Italien-Anleihen und damit auch zu höheren Kredit- bzw. Investitionskosten für Italiens Firmen führen.

Eine Italien-Staatsfinanzkrise wäre wegen der Größe Italiens automatisch eine Krise der Eurozone, zumal absolut gesehen italienische Staatsanleihen den größten nationalen Anteil an Gesamtbestand von staatlichen Bonds in der Eurozone haben. Damit droht mittelfristig das Risiko einer neuen Eurokrise, sobald Italien sein gerade noch bestehendes Investment Grade Rating bei den großen Rating-Agenturen verliert (bei S&P ist Italien allerdings noch zwei Stufen vom Absturz entfernt) oder falls eine Rating-Herabstufung der USA auch zu einer breiten Überprüfung der Staatsanleihe-Ratings in der Eurozone führt. Eine denkbare zweite Eurokrise wird durch das 750 Mrd. Euro EU-Kreditpaket vom Juli 2020 grundsätzlich nicht verhindert. Die Möglichkeit, Joint Eurobonds (JEB), also mit Gold und Devisenreserven teilbesicherte Euroanleihen, über einen von den Euro-Ländern getragenen JEB-Fonds (außerhalb der EU-Institutionen) aufzulegen, hat man bislang nicht genutzt. Ohne eine zweite Eurokrise dürfte der ökonomische Aufschwung in der Eurozone 2021 stärker als in den USA ausfallen. Entlastend für die USA, die Eurozone und Großbritannien sowie Deutschland wirkt bei der Staatsverschuldung die langfristige Absenkung der Nominalzinssätze seit etwa 1995 (vgl. Abbildung 1). Und weil auch die Realzinssätze in den OECD-Ländern gesunken sind, hat sich im Zeitraum 2005 bis 2020 abgesehen von den Schockjahren 2009 und 2020 kaum eine deutliche Wachstumsverlangsamung in den USA und der EU ergeben.

Da sich der Nominalzins nach der Fisher-Formel als Summe von Realzins plus erwarteter Inflationsrate ergibt, kann man bei Betrachtung von Nominal- und Realzins in den USA für die fünf Jahre vor dem Corona-Schock auf eine erwartete Inflationsrate von kaum 1 % schließen (ähnliche Überlegungen gelten für Großbritannien und die Eurozone), sodass auch die tatsächlichen Inflationsraten mittelfristig kaum ansteigen werden. Nach dem harten realwirtschaftlichen Corona-Schock von 2020 gilt das für die USA – und die Eurozone – erst recht.

Für die USA ist allerdings der 2020 erfolgte massive Anstieg der US-Arbeitslosenquote auf zwischenzeitlich mehr als 10 % ein erheblicher Schock, und zwar viel stärker noch als etwa der Anstieg der Arbeitslosenquo-

Tabelle 4
**Staatliche Schuldenquoten, ausgewählte Länder**
in % des BIP

| Bruttoschulden-position | 1990 | 1995 | 2000 | 2005 | 2010 | 2015 | 2019 | 2020[a] | 2021[a] |
|---|---|---|---|---|---|---|---|---|---|
| Japan | 64,30 | 95,90 | 137,89 | 176,62 | 207,68 | 231,34 | 237,38 | 251,91 | 247,63 |
| Italien | 101,33 | 119,36 | 109,02 | 106,55 | 119,18 | 135,26 | 134,77 | 155,54 | 150,43 |
| USA | | | | 65,45 | 95,48 | 104,79 | 108,98 | 131,07 | 131,90 |
| Frankreich | 35,58 | 56,10 | 58,88 | 67,38 | 85,26 | 95,58 | 98,55 | 115,42 | 116,38 |
| Spanien | 41,36 | 61,66 | 57,82 | 42,43 | 60,52 | 99,30 | 95,47 | 113,42 | 114,61 |
| Großbritannien | 28,56 | 43,74 | 36,79 | 39,58 | 74,56 | 86,92 | 85,41 | 95,73 | 95,78 |
| Deutschland | | 54,90 | 59,07 | 67,35 | 82,38 | 72,11 | 59,75 | 68,66 | 65,61 |
| Australien | 16,44 | 31,23 | 19,55 | 10,89 | 20,39 | 37,70 | 45,05 | 59,37 | 64,00 |
| Südkorea | 13,18 | 8,78 | 16,68 | 25,90 | 29,49 | 37,29 | 40,70 | 46,25 | 49,19 |
| Schweiz | 34,35 | 51,50 | 53,47 | 56,10 | 42,55 | 43,01 | 39,34 | 46,43 | 46,36 |

[a] Prognose.

Quelle: IWF (2020a), Fiscal Monitor April 2020, Datenbank.

te in Großbritannien oder Italien und Spanien im Corona-Schock-Jahr. Das liegt daran, dass die Krankenversicherung von Arbeitnehmern in den USA im Regelfall am Job hängt. Die hohen Arbeitslosenzahlen bedeuten von daher mitten in einer Pandemiesituation mit erhöhten Gesundheitsrisiken, dass Millionen Arbeitslose in den USA ohne Krankenversicherungsschutz sind; dem hat die US-Kongress-Gesetzgebung teilweise mit Blick auf COVID19-Erkrankungen bzw. Kostenübernahmezusagen entgegengewirkt. Dabei ist festzustellen, dass die Trump-Administration die Corona-Epidemie nur schwach bekämpft hat, etwa im Vergleich zu Deutschland: Wenn man die Sterbequote als Indikator nimmt, so gehören die USA zu den Top 10 in der Welt im ersten Halbjahr 2020 (Bretschger et al., 2020). Die Corona-Krise schwächt in den USA gerade die ökonomische Position vieler junger Menschen, etwa in der Altersgruppe der 20- bis 30-Jährigen: Erstmals seit den 1930er Jahren mit der Weltwirtschaftskrise wohnte 2020 wieder eine Mehrheit dieser Gruppe bei den Eltern (PEW Research Center, 2020).

## Struktureller Populismus-Druck in den USA

Der neue Populismus in den USA kann mit verschiedenen Ansätzen erklärt werden (Eichengreen, 2018; Welfens, 2019a), wobei eine veränderte – digitale – Gesellschaft und, im Zuge der Internetexpansion, eine politikrelevante Expansion radikaler Gruppen sowie ein geschwächter US-Grundkonsens beobachtbar sind. Was die Hinwendung großer Teile der US-Wählerschaft zum Populismus 2016 angeht, so ist diese Entwicklung offenbar wesentlich auf eine Kombination von langfristig enorm steigender Ungleichheit und politischer Frustration bei unteren Einkommensschichten zurückzuführen: Der Anteil der unteren Hälfte der Einkommensbezieher am Gesamteinkommen (Markteinkommen) ist in den USA von 20 % (1981) auf nur noch 13 % (2015) zurückgegangen, viel stärker als etwa in Westeuropa (Alvaredo et al., 2018). Auf die ärmeren Wählerschichten hob Trumps Wahlkampf 2016 wirksam ab, als er in seinen Reden immer wieder von den „forgotten men and women" sprach.

Zugleich zeigen Umfragedaten von Lindh und McCall (2018), dass die Mehrheit der befragten US-Bürger eine zunehmende Einkommensungleichheit beklagt, dabei jedoch eine relative Mehrheit nicht etwa die US-Politik als verantwortlich für die Umverteilung bzw. Sozialpolitik ansieht, sondern der Meinung ist, dass die großen US-Unternehmen die gewachsene Einkommensungleichheit korrigieren sollten. Diese Vorstellung ist jedoch ziemlich abwegig und unrealistisch, da in einer Shareholder Economy nicht zu erwarten ist, dass die Eigentümer Vorstands- bzw. Managergehälter quasi aus Gerechtigkeitserwägungen kürzen werden und die unteren Löhne der Ungelernten deutlich anheben werden (Welfens, 2019a; 2020a). Eine Möglichkeit wäre die Erhöhung des Mindestlohns. Es gibt zwar auch Bundesstaaten, die den Mindestlohn angehoben haben, aber er wurde auf Bundesebene über viele Jahre so festgesetzt, dass er real rückläufig war. Trumps Wahlsieg 2016 stützte sich wesentlich auf die untere Hälfte der Einkommensbezieher plus eine relativ kleine Gruppe sehr wohlhabender US-Bürger; letztere erwarteten vor allem Steuersenkungen und Deregulierungen.

Die Steuerpolitik von Trump hat keineswegs einen Ausbau der Sozial- oder Umverteilungspolitik zugunsten armer

Abbildung 1
**Langfristige Nominalzinssätze (Staatsanleihen) in den USA, der Eurozone, Deutschland und Großbritannien**

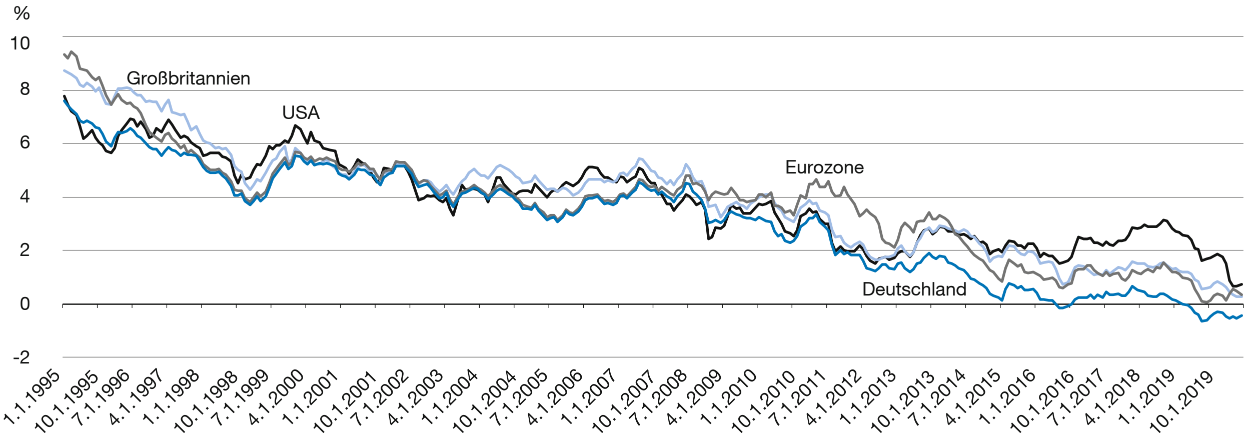

Quelle: eigene Darstellung, OECD (2020).

Schichten gebracht, vielmehr hat Trumps Steuerreformpolitik die Einkommensanteile der Top-Verdiener in den USA weiter erhöht; zudem ist der Anteil der US-Bürger ohne Krankenversicherungsschutz – noch vor dem Corona-Jahr 2020 – gegenüber der Obama-Administration von 11 % auf 13 % angestiegen (Welfens, 2019a, 2020a).

Zugleich gibt es aus technologischen Gründen und wegen des globalen Strukturwandels, der durch die hohen chinesischen Exporte bestimmter Gütergruppen angestoßen wurde, eine Tendenz hin zur Stagnation bzw. zum Sinken der Löhne der Geringqualifizierten (Breemersch et al., 2017). In den USA gelten eine relativ schwache Wettbewerbspolitik und unter der Trump-Administration auch die Steuerreform als Treiber von wachsender Ungleichheit (Stiglitz, 2018). Eine IWF-Studie (Jaumotte et al., 2008) hat gezeigt, dass die realwirtschaftliche Globalisierung zu geringeren Pro-Kopf-Einkommensunterschieden zwischen den Ländern geführt hat, dass aber die Finanzmarkt-Globalisierung die Einkommensunterschiede zwischen den Ländern erhöht; verstärkt werden diese Unterschiede noch durch die Expansion der Informations- und Kommunikationstechnik (IKT). Die Finanzmarkt-Globalisierung brachte weltweit verminderte Realzinssätze und damit leichtere Kreditaufnahmemöglichkeiten für die meisten Individuen; solche mit relativ hohen Einkommen – in der Regel qualifizierte Arbeitnehmer – und Vermögende, die entsprechend leicht Kreditsicherheiten für Bankkredite anbieten können. Ungelernte sind von daher die Verlierer der Globalisierung, zumal die Expansion des IKT-Sektors technologiebedingt auch noch eine verstärkte Nachfrage nach Qualifizierten mit sich bringt. Die Reallöhne der Geringqualifizierten gerade in den USA haben seit den 1980er Jahren in etwa stagniert, hinzu kommt noch, dass viele US-Bürger keine oder nur eine sehr minimale Krankenversicherung haben und die Lebenserwartung in den USA geringer als in Westeuropa ist.

## Corona-Schock verstärkt ökonomische Ungleichheit

Die massive Corona-Rezession in den USA bringt für Arbeitslose und in Konkurs gehende Selbstständige in vielen Fällen einen Verarmungsimpuls, aber jenseits des Rezessionsschocks gibt es auch weitere Impulse für Ungleichheit. Die Tendenz zu erhöhter Ungleichheit in den USA – und einigen anderen OECD-Ländern – wird durch die Corona-Schocks noch verstärkt, da wegen der Epidemie-Bedrohung die Expansion des IKT-Sektors zugenommen hat, der überwiegend qualifizierte Arbeitnehmer beschäftigt. Im Corona-Schockjahr 2020 haben viele Unternehmen in vielen Ländern z. B. verstärkt auf Home-Office-Wertschöpfung als Strategie zur Minderung von Infektionsrisiken und zur Fortsetzung der Produktion gesetzt; eine auch nach 2020 sich wohl fortsetzende digitale Expansionstendenz. Das bedeutet ökonomisch gesehen für 2020 auch, dass der digitale Kapitalstock deutlich zugenommen hat und zwar zu Grenzkosten nahe null (z. B. Software-Nutzung).

Dieser quasi-exogene digitale Angebotsschock bedeutet in einer offenen Volkswirtschaft gemäß dem Rybczynski-Theorem, dass die digital-intensive Produktion zunimmt, während die anderen Produktionsbereiche absolut abnehmen. Da digital verstärkte Wertschöpfung mit einer erhöhten Produktinnovationsrate und mehr Preisdifferenzierung – bei faktischen durchschnittlichen Preiserhöhungen – einhergeht, kann man zudem das Stolper-

Samuelson-Theorem nutzen, das besagt: Wenn es zu einer exogenen Preiserhöhung eines Produkts in einer offenen Volkswirtschaft kommt, dann wird die Entlohnung des Produktionsfaktors relativ ansteigen, der bei dessen Produktion relativ intensiv eingesetzt wird. Also läuft auch das wieder auf einen Anstieg der Lohnprämie für Qualifizierte hinaus und bedeutet für die USA wie für Westeuropa und Japan, dass die ökonomische Ungleichheit Corona-bedingt ansteigt (Welfens, 2020b).

Dem kann man etwa durch verstärkte Weiterbildungsaktivitäten von Ungelernten grundsätzlich entgegentreten, aber die USA gaben in den 15 Jahren nach 2002 nach OECD-Angaben staatlicherseits nur etwa 0,04 % (Großbritannien 0,01 %) des BIP für Weiterbildung aus; in der Schweiz waren es – bei Vollbeschäftigung – um 0,2 %, in Deutschland um 0,25 % und im EU-Spitzenreiterland Dänemark sogar 0,6 %. Dabei zeigt eine Studie aus den Niederlanden (Fouarge et al., 2013), dass die soziale Rendite von Weiterbildung für Qualifizierte und Ungelernte in etwa gleich hoch ist – bei allerdings relativ schwacher Weiterbildungsmotivation von Ungelernten. Hier bestehen ernste Herausforderungen.

### Transatlantische und Globale Politikperspektiven

Auch wenn Donald Trump die Präsidentschaftswahl nicht wiedergewinnen sollte, bleiben die USA eine seit 2016 deutlicher als bisher polarisierte Gesellschaft – ökonomisch und politisch. Diese Polarisierung schwächt mittel- und langfristig die Fähigkeit der USA zur internationalen Führung. Die EU, durch Brexit allerdings geschwächt, ist von daher gefordert, ihrerseits mehr politische Führung zu leisten und auch die Rolle internationaler Organisationen bzw. des Multilateralismus stärker zu verteidigen als bisher, sofern die Weltwirtschaftsordnung im 21. Jahrhundert stabil bleiben soll. Dabei müssten fast alle OECD-Länder an einer Fortsetzung der regelbasierten Weltwirtschaftsordnung interessiert sein. Mit der aus ökonomischer Sicht sonderbaren Brexit-Entscheidung, die man als Form eines britischen (oder englischen) Populismus ansehen kann (Welfens, 2018), hat neben den USA unter Trump auch die alte globale Führungsmacht Großbritannien den nach 1945 entstandenen westlichen Konsens in internationalen Wirtschaftsfragen verlassen.

Dass sich ausgerechnet das freihändlerisch eingestellte Großbritannien – unter den Premiers May und Johnson mit dem Slogan „Global Britain" regierungsseitig plakativ sichtbar gemacht – nach dem EU-Referendum 2016 verstärkt auf die Seite des offen protektionistisch auftretenden US-Präsidenten Trump stellte, ist auch eine Widersprüchlichkeit britischer Politik. Trump hat mit einer aggressiven Handelspolitik nicht nur gegenüber China, sondern auch gegenüber den OECD-Partnerländern aufgewartet und mit dem Austritt aus dem UN-Klimaschutzabkommen von Paris ein negatives Kooperationssignal beim Klimaschutz gegeben.

Der Westen dürfte durch den Corona-Schock gegenüber China bzw. Asien ökonomisch an Einfluss verloren haben, da der Neustart der Wirtschaft in China und den ASEAN-Ländern – trotz der anhaltenden Handelskonflikte mit den USA auch 2020 – offenbar besser gelingt als in den USA plus EU plus Großbritannien. Für den längerfristigen ökonomischen Aufschwung im Westen bringt neben der verstärkten digitalen Expansion immerhin die starke Absenkung der Realzinssätze einen für die Investitionsquote, aber auch die Innovationsdynamik wesentlichen Impuls.

Wenn man den Schumpeter-Wachstumsansatz von Aghion und Howitt (2007) betrachtet, so bedeutet ein verminderter Realzinssatz, dass für Innovatoren der Gegenwartswert schumpeterianisch erhöhter künftiger Gewinne etwa aus Patenten wachstumsförderlich ansteigt. Von daher könnte nach 2020 auch in den OECD-Ländern mittelfristig ein starker Aufschwung einsetzen. Dieser könnte wiederum, wie auch die ökonomischen Expansionsperspektiven Asiens, durch einen global wachsenden Nationalismus (De Bolle und Zettelmeyer, 2019) geschwächt werden, der typischerweise mit Protektionismus einhergeht. Die USA stehen im Corona-Jahr vor der bitteren Erkenntnis, dass das US-Wirtschafts- und Politiksystem ebenso wie das Gesundheitssystem geschwächt ist – und für Großbritannien gilt das mit geringen Einschränkungen ebenso.

Dieser Befund kann durchaus als positives Argument für die Festigung und Weiterentwicklung der Sozialen Marktwirtschaft in Deutschland und der EU eingeordnet werden; bei sicherlich hohem Reformdruck in EU-Ländern wie Italien und Frankreich, wobei letzteres im Corona-Schock beim Gesundheitssystem unerwartet schwach im EU-Vergleich abgeschnitten hat. Es bleibt abzuwarten, ob die deutsche EU-Ratspräsidentschaft das Thema Festigung und Export der Sozialen Marktwirtschaft mit Ernst, Energie und Augenmaß aktiv aufnehmen wird. Sieht man von den staatlichen Gesundheitsausgaben ab, so stehen die Sozialausgaben der USA für kaum 2 % des BIP, während die entsprechenden Sozialausgabenquoten in der Schweiz 8 %, in den Niederlanden und Großbritannien etwa 12 %, in Deutschland 14 % sowie in Frankreich und Italien knapp 20 % betragen.

Die Transatlantischen EU-USA-Kooperationsmöglichkeiten dürften deutlich ansteigen, falls am 3. November nicht Präsident Trump die US-Präsidentschaftswahl gewinnt; dies gilt zumal für die Klimaschutzpolitik. Hier wäre ein internationaler Ausbau des Handels mit $CO_2$-Emissionszertifikaten im G20-Raum gerade für die Post-Corona-Rezessions-Aufschwungphase möglich, was eine global effiziente kostenminimale Klimapolitik (Welfens, 2019b) brächte.

Die EU-Kommission hat wie die Bundesregierung in Deutschland im Sommer 2020 betont, dass ein nachhaltiger Aufschwung mit Fokus gerade auch auf Digitalisierung, mehr Klimaschutz und Modernisierung der Gesundheitssysteme angestrebt sei. Für diese Expansionsrichtung gibt es einige gute Argumente, wobei der Staat sich von selektiver Industriepolitik mit sektoralen Sonderinterventionen eher fernhalten sollte; Ausnahmen sind nur in innovationsstarken Bereichen denkbar, sofern es positive externe Effekte national oder international gibt. Transatlantisch werden mittelfristig auch Konfliktfelder in der Wirtschaftspolitik bleiben, etwa die Frage der Besteuerung von digitalen Transaktionen. Ein neuer transatlantischer ordnungspolitischer Dialog mit Fokus auf mehr Soziale Marktwirtschaft in den USA könnte Teil eines Neustarts der transatlantischen Politikkooperation nach 2020 sein, der den Westen und die Weltwirtschaft insgesamt stärken könnte. Hierfür gilt es geeignete Formate und Projekte zu entwickeln, wobei sowohl Europa als auch die USA von einem neuen transatlantischen Zukunfts-Dialog einen Nutzen haben können.

## References

[CR1] Aghion P, Howitt P (2007). Capital, innovation, and growth accounting. Oxford Review of Economic Policy.

[CR2] Alvaredo, F., L. Chancel, T. Piketty, E. Saez und G. Zucman (2018), World Inequality Report 2018, *World Inequality Report*, https://en.unesco.org/inclusivepolicylab/sites/default/files/publication/document/2018/7/wir2018-full-report-english.pdf (27. Oktober 2020).

[CR3] Breemersch, K., J. Damijan und J. Konings (2017), Labour Market Polarization in Advanced Countries: Impact of Global Value Chains, Technology, Import Competition from China and Labour Market Institutions, *OECD Social, Employment and Migration Working Papers*, Nr. 197, OECD Publishing.

[CR4] Bretschger, L., E. Grieg, P. J. Welfens und T. Xiong (2020), COVID-19 Infections and Fatalities Developments: Empirical Evidence for OECD Countries and Newly Industrialized Economies, *EIIW Discussion Paper*, Nr. 277, EIIW/University of Wuppertal, https://uni-w.de/d9lil (25. September 2020). 10.1007/s10368-020-00487-x PMC7556566 40477202

[CR5] CBO (2020), The Budget and Economic Outlook: 2020 to 2030, Congressional Budget Office, https://www.cbo.gov/publication/56073 (25. September 2020).

[CR6] De Bolle, M. und J. Zettelmeyer (2019), Measuring the Rise of Economic Nationalism, *Petersen Institute for International Economics Working Paper*, 19-15, Washington DC, https://www.piie.com/sites/default/files/documents/wp19-15.pdf (25. September 2020).

[CR7] Eichengreen, B. (2018), *The Populist Temptation - Economic Grievance and Political Reaction in the Modern Era*, Oxford University Press.

[CR8] Fourage D, Schils und T, De Grip A (2013). Why Do Low-Educated Workers Invest Less in Further Training?. Applied Economics.

[CR9] Frankel, J. (2006), Twin Deficits and Twin Decades, in R. Kopcke, G. Tootell und R. Triest (Hrsg.), *The Macroeconomics of Fiscal Policy*, MIT Press.

[CR10] IWF (2019), Article IV Consultation, United States, *IMF Country Report*, 19/17, Juni.

[CR11] IWF (2020a), IMF Fiscal Monitor April 2020, https://www.imf.org/en/Publications/FM/Issues/2020/04/06/fiscal-monitor-april-2020 (25. September 2020).

[CR12] IWF (2020b), Article IV Consultation, United States, *IMF Country Report*, Nr. 20/241, August.

[CR13] IWF (2020c), World Economic Outlook Update, Juni.

[CR14] Jaumotte, F., S. Lall und C. Papageorgiou (2008), Rising income inequality: Technology, or trade and financial globalization, *IMF Working Paper*, WP/08/185.

[CR15] Lindh, A. und L. McCall (2018), Reconsidering the Popular Policy of Redistribution: Preferences for Reducing Economic Inequality in the US. Paper presented at the Expert Group Meeting on “New research on Inequality and its Impacts” at United Nations Headquarters in New York, 12.-13. September.

[CR16] OECD (2020), Main Economic Indicators - complete database, monatliche Daten von 1. Januar 1995 bis 1. Juni 2020, 10.1787/data-00052-en (17. August 2020).

[CR17] PEW Research Center (2020), A majority of young adults in the U.S. live with their parents for the first time since the Great Depression, Pew Research Center, September.

[CR18] Stiglitz J E (2018). A Rigged Economy. Scientific American.

[CR19] Welfens, P. J. J. (2018), *Brexit aus Versehen: Europäische Union zwischen Desintegration und neuer EU*, 2. Aufl., Springer.

[CR20] Welfens, P. J. J. (2019a), *The Global Trump: Structural US Populism and Economic Conflicts with Europe and Asia*, Palgrave.

[CR21] Welfens, P. J. J. (2019b), *Klimaschutzpolitik - Das Ende der Komfortzone*, Springer.

[CR22] Welfens, P. J. J. (2020a), *Trump global*, Springer.

[CR23] Welfens, P. J. J. (2020b), *Corona-Weltrezession*, Springer.

